# Prevalence of stunting and associated factors among public primary school pupils of Bahir Dar city, Ethiopia: School-based cross-sectional study

**DOI:** 10.1371/journal.pone.0248108

**Published:** 2021-04-12

**Authors:** Getasew Mulat Bantie, Amare Alamirew Aynie, Kidist Hailu Akenew, Mahlet Tilahun Belete, Eyerusalem Teshome Tena, Genet Gebreselasie Gebretsadik, Aynalem Nebebe Tsegaw, Tigist Birru Woldemariam, Ashenafi Abate Woya, Amare Alemu Melese, Agumas Fentahun Ayalew, Getenet Dessie

**Affiliations:** 1 Faculty of Community Health, Alkan Health Sciences, Business and Technology College, Bahir Dar, Ethiopia; 2 Department of Nursing, GAMBY College of Medical Sciences, Bahir Dar, Ethiopia; 3 Department of Statistics, Bahir Dar University, Bahir Dar, Ethiopia; 4 Department of Medical Laboratory, University of Gondar, Gondar, Ethiopia; 5 School of Public Health, College of Health Sciences, Woldia University, Woldia, Ethiopia; 6 Department of Nursing, Bahir Dar University, Bahir Dar, Ethiopia; Universitas Airlangga, UNITED STATES

## Abstract

**Background:**

Stunting is a well-established child-health indicator of chronic malnutrition, which reliably gives a picture of the past nutritional history and the prevailing environmental and socioeconomic circumstances.

**Objective:**

To investigate the prevalence of stunting and associated factors among public primary school children of the Bahir Dar city.

**Method:**

A cross-sectional study was carried out from March to June 2019. Data were coded and entered into the Epi-Data and exported to SPSS version 23 software. The pupil was stunted if the height- for-age was ≤ -2 SDs from the median growth standards according to the WHO. A descriptive summary was computed using frequency, percent, mean, median and standard deviation. A simple logistic regression model was fitted to identify associated factors between the independent variables and the dependent variable at a 95% confidence interval (CI) and p-value <0.05.

**Results:**

370 primary school pupils were included in the study with the mean age of 10.15 (± 2.23 SD) years. 51.6% of the pupils were females. The total prevalence of stunting was 15.13% (95%CI; 11%, 19%). The burden of stunting was higher in the age group of 11 years and older. Pupil’s age older than 11 years (AOR = 15. 6; 95%CI; 3.31, 73.45; p-value < 0. 001) and male sex (AOR = 7. 07; 95%CI: 2.51, 19.89; p-value < 0. 0002) were significantly associated with stunting.

**Conclusion:**

The prevalence of stunting was relatively lower than the regional estimated stunting level. Older age and male sex were significantly associated with stunting.

## Introduction

School children pass through great physical and mental changes, which affect both their growth and school performance [1], and nutrition is one of the many key factors affecting mental development of children [2].

Undernutrition is a condition that results from insufficient intake of energy and nutrients to meet an individual’s needs to maintain good health [3]. Undernutrition is widespread among school children in low income countries, particularly in Africa, and their nutritional status often worsens during their school years. Undernutrition greatly affects both the cognitive and physical development of school age children [4], and increases susceptibility to infection [5].

Undernutrition-related health challenges in school-age children are among the most frequent reasons for low school enrolment, high absenteeism, early dropout, poor academic performance, delayed cognitive development, short stature, reduced work capacity, and poor reproductive performance [4]. Stunting is the collective impact of glitches in socioeconomic status and persistent infections. It originates from long-term nutritional deprivation, inadequate childcare and poor environmental and social conditions [6]. It affects 165 million children worldwide, 90% of whom live in Africa and Asia [7]. Studies revealed that different countries had different figures of stunting. Globally, the prevalence of stunting among school age pupils ranges from 20 to 80% [8,9].

According to a study done in rural Ethiopia, the extent of stunting in school-age children ranges from 26.5% to 42.7% [10]. However, in urban school-age children the stunting level ranged from 5.4% to 29.2% [11]. But these reports are outdated and could not show the current situations of the stunting level.

Factors such as child age, family size, mother’s education, father’s occupation, and child’s immunization, public taps, tea drinking habit, anemia, born to a working and older mother, didn’t use a bed net, and illness of the child within the last 2 weeks have been associated with stunting [12–14].

The motive for conducting this study was to assist in the achievement of sustainable developmental goals (SDGs) through determining the stunting level and identifying the associated factors among pupils of public primary schools. These are the dragging risk factors for school dropout and may impact the target of quality primary education for all (SDGs-4) [15]. Furthermore, Previous studies were targeting on under-five stunting, and the stunting among primary school pupils were the neglected one. The other pushing reason for this study was the lack of data on the health of public primary school pupils, and the growing motives of governments and Aid agencies in the correlation of health and education.

## Methods

### Study design, setting and period

A school-based cross-sectional study was conducted among Bahir Dar city public primary school pupils from March 10 to June 10, 2019. Bahir Dar city is located 565 km away from Addis Ababa, the capital city of Ethiopia Bahir Dar city is the second biggest city in Ethiopia, next to Addis Ababa [16]. Bahir Dar city is the capital city of the Amhara regional state, where the higher prevalence of stunting was observed [17]. The city includes six administrative units or sub-cities. The total population of the city is estimated to be 249,851 (estimated population and household survey of 2017/18) (124,426 females and 125,425 males) [18]. In the city, there are 19 public primary schools (1 up to 8 graded) [19].

### Population, sample size determination, and sampling procedure

**Fig 1:**
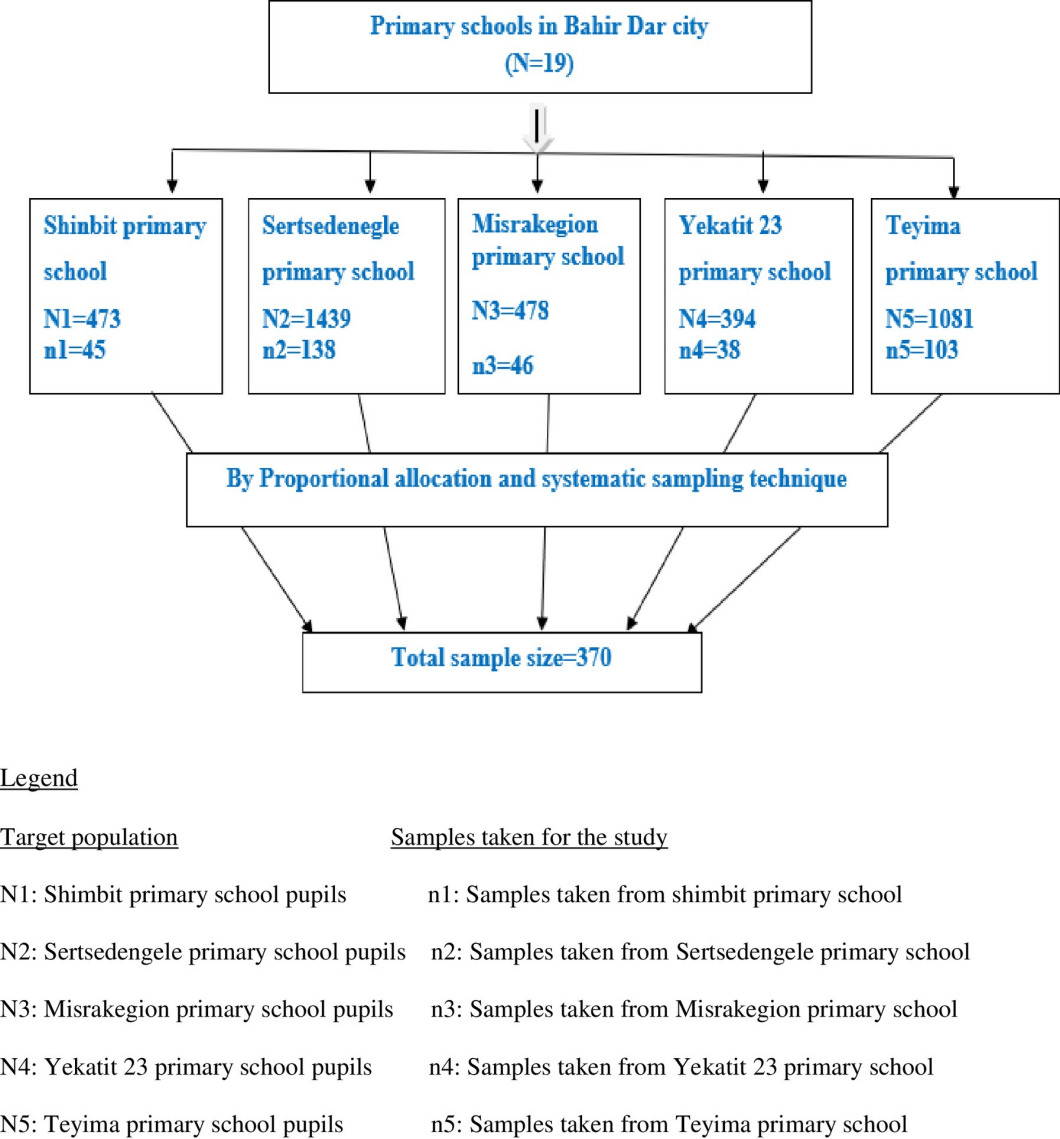


The source population for this study consisted of all pupils attending primary schools of the Bahir Dar city. The study population for this study was pupils attending the primary schools selected for the city. Those who were drop outs or absent during the data collection period were excluded. The sample size was determined using a single population proportion formula by considering: 95% confidence level, 5% margin of error, with the proportion of stunting of 18.3% in Bahir Dar city [20]. Taking the design effect 1.5 and 10% non-response rate, and considering the correction formula (N<10, 000), the final sample size was 375. Five primary schools were recruited from the total of 19 primary schools (grades 1 to 8) by lottery method. From the five selected schools, primary cycle (1 up to 4 graded) pupils were randomly recruited for the study. Samples were proportionally assigned to each of the selected primary schools based on the number of primary cycle students. Thus, 38, 45, 46, 103, and 138 pupils were selected from Yekatit 23, Shimbit, Misrakegion, Teyima, and Sertsedenegle primary schools, respectively, using the school attendance sheet via systematic random sampling technique [Fig 1].

### Data collection procedure

Data collection tools were initially prepared in English then translated to the local Amharic language (attached in Supporting information). Six nurses with Bachelor’s degree (four for data collection and two for supervision) were recruited. Two days training was given for data collectors and supervisors by the principal investigator about the objective of the study. Socio-demographic characteristics, health care characteristics, maternal awareness on dietary diversification, and household food security related data were collected from the parents using a pre-tested structured interviewer-administered questionnaire. Anthropometric data such as weight, height, and body mass index were taken from each study participant (pupil) by removing all clothes and shoes except sensitive wears like pants and shirts and the privacy was maintained. Measuring devices used were the ones distributed to the health institutions by Ethiopian ministry of health.

After portable anthropometric measurements were taken, the parents were invited to each respective school and were interviewed face to face based on the objective of the study. Each questionnaire had an information sheet attached with instructions to ensure that all respondents received the same directions and an informed consent form for respondents to read and sign if they agreed to be part of the study.

### Study variables and measurement

#### Stunting

The pupil was classified as stunted if the height- for-age was ≤ -2 SDs from median growth standards of WHO [21].

#### Underweight

The pupil was classified as underweight if the weight- for-age was ≤ -2 SDs from median growth standards of WHO [21].

#### Wasting

The pupil was classified as wasted if the weight- for-height was ≤ -2 SDs from median growth standards of WHO [21].

#### Dietary diversity

Is the number of various foods or food groups consumed over a given reference period [22]. In this study, dietary diversity was achieved (responded ‘yes’) when the pupil consumes at least three dietary groups (cereals, vegetables, and animal products) per day.

#### Food security

A condition that all parents of the pupil access safe, sufficient, and nutritious food all times to meet their dietary needs [23].

#### Ill pupil

In this study, a pupil was considered ‘ill’ if he/she was ill within the last two weeks of the data collection period.

### Quality control

The principal investigator (PI) trained the data collectors and the project investigators, who supervised the data collectors, on the goals of the project and on data quality. The enumerators and the project investigators carried out pre-test activities in a nearby town (Woreta) in five percent of the total sample size before the actual data collection. Then, vague sentences were explicitly rephrased. The internal consistency (Cronbach alpha) level of the pretest of stunting was 0.84.

Calibration of the digital weight scale (to zero) and cross-checking it using a pre-known weight material before weighing each pupil. The height of the pupil was measured after putting the pupil on the vertical wooden height measuring board. The pupil of the school was standing upright in the middle of the board. The pupil’s head, shoulders, buttocks, and heels are touching the measuring board. The height of the pupil was recorded to the nearest 0.1 cm. At the end of every data collection day, the project investigators examined each questionnaire and gave pertinent feedback to the enumerators.

### Data processing and analysis

The collected data were checked for completeness and consistency. The data were cleaned, coded, and entered to EPI- Data software. The analysis was done using SPSS (Version 20) and Anthro-plus software. Descriptive statistics were computed, and the chi-square assumptions were assessed to check the presence of the relationship between two categorical variables. The model fitness was checked using Hosmer and Lemeshow goodness of fit (P> 0.05). A simple logistic regression model was used to identify the association between the explanatory variables and stunting. Adjusted Odds ratio (AOR) with 95% CI (confidence interval) was used to measure the strength of association between explanatory variables and the stunting.

The chi-square (X^2^) test was checked and a p-value < 0.05 at x^2^ test was considered as having relationship between predictor variables and stunting and these variables were run in to bivariate regression analysis. Then, predictor variables having a p-value < 0.20 at bivariate regression analysis were taken into a multivariable logistic regression analysis to see associations between dependent and independent variables. The backward logistic regression method was used, and variables with a p-value of < 0.05 at multivariable analysis were considered as statistically significant predictors of stunting.

### Ethical considerations

Ethical approval was obtained from GAMBY Medical and Business College, Research and Publication Office with the reference number of GC-221/2011. Before the beginning of data collection, permission letter was obtained from the Amhara National, Regional Institute of Public Health prior to the data collection period. The support letter was obtained from Bahir Dar city health and education department. The school directors were informed about the purpose of the study that it will contribute to the health needs of the students. For pupils of 7–12 years old, the written consent was taken from his/her parents/guardians. For pupils of 13–17 years old, consent was secured from them only with parental/guardian permission. However, for pupil’s older than 17 years, written consent was secured solely from them. The objective of the study was clarified to the respondents and they were notified that they have the right to refuse or terminate the study at any point of the interview. The written consent and the data collection tools were documented and kept confidential in a secure place. Stunted pupils’ families were extensively advised on the importance of a balanced diet and they were referred to consult the nearby health institution for technical support for the at least to halt the further complication of stunting.

## Results

### Sociodemographic characteristics of the study participants

A total of 370 primary school pupils was recruited for the study with a response rate of 98.6%. The mean age of the respondents was 10.15 (± 2.23 SD) years. Twelve percent of female pupils were stunted compared to 19% among male pupils. Thirty-five percent of pupils with 11 and above family size were stunted compared to 14% among five and lower family size. Twenty percent of the merchant parents’ pupil were stunted compared to 11.1% of housewife. Eighteen percent of the non-formal education attended parents’ pupil were stunted compared to 11% of secondary and above educated. Twenty percent of the 500 birrs and lower monthly earned parents’ pupil were stunted compared to 14% of 500 birrs and above. About 87% of the pupil were under-weight while nearly 41% of the pupil were wasted [Table 1].

**Table 1:** The relationship of socio-demographic characteristics and stunting among Bahir Dar city public primary school pupils, North West Ethiopia, 2019.

Variable	Stunted	Not-stunted	Total	X^2^, P-value
	N (%)	N (%)	N (%)	
Age of the pupil (in years)				
6–8	7 (6.2)	90 (93.8)	97 (26.2)	16.37, 0.001
9–10	14 (10.8)	116 (89.2)	130 (35.1)	
≥ 11	35 (24.5)	108 (75.5)	143 (38.7)	
Sex of the pupil				
Female	22 (11.5)	169 (88.5)	191 (51.6)	4.02, 0.045
Male	34 (19.0)	145 (81.0)	179 (48.4)	
Religion				
Orthodox	49 (14.7)	283 (85.3)	332 (89.7)	----
Islam	7 (22.6)	24 (77.4)	31 (8.4)	
Catholic	0 (0)	2 (100)	2 (0.6)	
Protestant	0 (0)	5 (100)	5 (1.3)	
Household family size				
1–5	27 (13.6)	172 (86.4)	199 (53.8)	6.56, 0.038
6–10	22 (14.6)	129 (85.4)	151 (40.8)	
11–15	7 (35)	13 (65)	20 (5.4)	
Maternal occupation				
Merchant	9 (20.0)	36 (80.0)	45 (12.2)	3.90, 0.272
Government employee	6 (12.5)	42 (87.5	48 (12.9)	
Daily laborers	26 (18.3)	116 (81.7)	142 (38.4)	
Housewife	15 (11.1)	120 (88.9)	135 (36.5)	
Maternal education				
Can’t read and write	18 (15.5)	98 (84.5)	116 (31.3)	2.28, 0.683
Can read and write only^¥^	23 (17.8)	106 (82.2)	129 (34.9)	
Primary school	8 (12.7)	55 (87.3)	63 (17.1)	
Secondary and above	7 (11.3)	55 (88.7)	62 (16.7)	
Monthly income (in Birrs)				
<500	12 (20.3)	47 (79.7)	59 (15.9)	1.50, 0.682
500–1000	13 (14.1)	79 (85.9)	92 (24.9)	
1001–1500	8 (13.6)	51 (86.4)	59 (15.9)	
> 1500	23 (14.4)	137 (85.6)	160 (43.3)	
Body mass index				
Under-weight	46 (14.3)	275 (85.7)	321 (86.8)	2.64, 0.267
Normal	9 (19.1)	38 (80.9)	47 (12.7)	
Over-weight	1 (50)	1 (50)	2 (0.5)	
Mid upper arm circumstance				
Wasted	27 (17.9)	124 (82.1)	151 (40.8)	1.49, 0.221
Normal	29 (13.2)	190 (86.8)	219 (59.2)	

^¥^ Mothers who had no exposure of formal education in the school like primary, secondary, preparatory), but they tried to read any written word/phrase or write their name or other thing.

### Household food security and dietary diversification characteristics

About twenty-two percent (22.2%) of the stunted children were from parents who were worried about not having enough household food and sharing food when there is no enough food. About one-fifth (19%) of the stunted child’s parents thought their family did not get enough food [Table 2].

**Table 2:** The relationship of household food security, dietary diversification, and stunting among Bahir Dar city public primary school pupils, North West Ethiopia, 2019.

Variable	Stunted	Not-stunted	Total	X^2^, p-value
	N (%)	N (%)	N (%)	
You faced food shortage				
Yes	22 (15)	125 (85)	147 (39.7)	0.005, 0.94
No	34 (15.2)	189 (84.8)	223 (60.3)	
You worried about not having enough* household food				
Yes	35 (22.2)	123 (77.8)	158 (42.7)	10.57, 0.001
No	21 (9.9)	191 (90.1)	212 (57.3)	
Frequency of worrying due to fear of not having enough household food				
Always	10	27	37	8.115, 0.017
Usually	7	57	64	
Sometimes	18	39	57	
Your action when your family gets a shortage of food				
Share with relatives	12 (22.2)	42 (77.8)	54 (14.6)	3.69, 0.448
Debt	14 (13.2)	92 (86.8)	106 (28.7)	
Work hard	20 (16.1)	104 (83.9)	124 (33.5)	
Do nothing	4 (9.3)	39 (90.7)	43 (11.6)	
Asking for donation	6 (14)	37 (86)	43 (11.6)	
You think your family member didn’t get enough food*				
Yes	23 (19)	98 (81)	121 (32.7)	2.10, 0.147
No	33 (13.3)	216 (86.7)	249 (67.3)	
Reason for food shortage				
Divorce	4 (10.8)	33 (89.2)	37 (25.2)	7.58, 0.106
Alcohol abuse	2 (8)	23 (92)	25 (17.0)	
Income shortage	14 (24.1)	44 (75.9)	58 (39.5)	
Lack of national peace	0 (0)	13 (100)	13 (8.8)	
Economic instability	2 (14.3)	12 (85.7)	14 (9.5)	
The mother participated in child feeding				
Yes	44 (13.7)	277 (86.3)	321 (86.8)	3.84, 0.051
No	12 (24.5)	37 (75.5)	49 (13.2)	
Heard about variety of food				
Yes	52 (14.8)	299 (85.2)	351 (94.9)	0.546, 0.46
No	4 (21.1)	15 (78.9)	19 (5.1)	
Sources of information				
Health care provider	22 (13.8)	138 (86.3)	160 (45.6)	5.25, 0.154
Family members	5 (8.1)	57 (91.9)	62 (17.7)	
Television	17 (23.0)	62 (77.0)	79 (22.5)	
Radio	8 (21.4)	42 (78.6)	50 (14.2)	
Variety of food is good for your children				
Yes	55 (15.1)	309 (84.9)	364 (98.4)	0.011, 0.92
No	1 (16.7)	5 (83.3)	6 (1.6)	
Place you will take when your child gets sick				
Health institution	39 (14.8)	225 (85.2)	264 (71.4)	1.49, 0.474
Traditional medicine	4 (10.5)	34 (89.5)	38 (10.3)	
Holly water	13 (19.7)	55 (80.3)	68 (18.4)	
Any chronic disease** in the family				
Yes	6 (17.6)	30 (83.3)	36 (16.7)	0.073, 0.78
No	50 (15.0)	284 (85.0)	334 (83.3)	
The current health status of this pupil				
Ill	4 (15.0)	20 (85.0)	24 (6.5)	0.047, 0.83
Healthy	52 (16.7)	294 (83.3)	346 (93.5)	

Enough food

* (eating food at least three times per day); chronic disease

** (diabetes mellitus, hypertension and/or asthma).

### The prevalence of stunting among primary school pupils

This study revealed that 15.13% of the pupils of the city public primary school was stunted. The highest level of stunting happened at Sertsedengel primary school (41.1%), followed by Teyima (39.3%), Shimbet (8.9%), Yekatit 23 (7.1%), and Misrakeghion primary schools (3.6%), respectively. The highest proportion of stunting in Sertsedengel primary school could be as this is found at the center of the city, where many of the poor are living, it may expose them for a higher level of stunting.

### Factors associated with stunting among primary school children

With the multivariable logistic regression model, age and sex of the pupil were significantly associated with stunting at a p-value of 0.05.

Accordingly, those primary school children in the age group of 11 years and older were about 15 times, (AOR = 15. 6: 95%CI: 3.31, 73.45) more likely to be stunted compared to the age group of 6–8 years. Similarly, the odds of stunting among male pupils were about 7 times (AOR = 7. 07; 95%CI: 2.51, 19.89) higher compared to those female pupils [Table 3].

**Table 3:** Simple logistic regression analysis on factors associated with stunting among Bahir Dar city public primary school pupils, North West Ethiopia, 2109.

Variable	Stunting		COR (95%CI)	AOR (95%CI)	P-value
	Yes (N)	No (N)			
Age of the pupil (in years)					
6–8	7	90	1.00	1.00	
9–10	14	116	1.55 (0.6,4.0)	4.44 (0.88,22.34)	0.070
≥ 11	35	108	4.17 (1.76,9.83)	15.6 (3.31,73.45)	0.001
Family size					
1–5	27	172	1.00	1.00	
6–10	22	129	1.08 (0.59,1.99)	1.83 (0.72,4.67)	0.199
11–15	7	13	3.43 (1.26,9.36)	2.95 (0.43,19.81)	0.266
Worrying due to fear of not having enough household food					
Yes	35	123	2.58 (1.44, 4.65)	1.21 (0.43,3.43)	0.717
No	21	191	1.00	1.00	
Frequency of worrying due to fear of not having enough household food					
Always	10	27	1.00	1.00	
Usually	7	57	0.33 (0.11,0.96)	0.45 (0.13,1.25)	0.116
Sometimes	18	39	1.24 (0.49,3.11)	1.75 (0.64,4.79)	0.274
Sex of the pupil					
Female	22	169	1.00	1.00	
Male	34	145	1.80 (1.00,3.21)	7.07 (2.51,19.89)	0.0002

COR: Crude Odds Ratio; AOR: Adjusted Odds Ratio; P-value; probability value.

## Discussion

The prevalence of stunting was 15.13% (95%CI; 11, 19). This finding was lower than the EDHS-2016 report, 38% [24]. Similarly, the current study finding is lower than other indigenous studies of Jimma Zone, 24.1% [25], Gondar town, 46.1% [26], Humbo district, 57% [27], Mecha District, 37.9% [28], Arba Minch city, 41.9% [29], and southern Ethiopia, 28% [30]. This difference might be due to, the current study was done in the Bahir Dar city, where the health information, dissemination and communication extensively distributed to the communities, and the households might be entirely aware of the nutrition related issues via different mass media, which in fact might allow the pupils get regular balanced diet as per their parents’ income permit. However, this study finding was higher compared to studies in Sudan, 3.8% - 7.1% [31,32], Nigeria, 10.5% [33], Iran, 3.7% [34], China, 1.0% - 4.7% [35–37], Pakistan, 8% [38], and Colombia, 19.8% [39]. The possible explanation for this discrepancy might be the difference in study settings, social norms, lifestyles, health care set up, and health information dissemination. Pupil’s age of 11 years and older were about sixteen times more likely to be stunted than the age group of 6 up to 8 years. This finding was supported by studies in Ethiopia [7,12,14,40], Tanzania [41], Nigeria [42,43], Iraq [44], Pakistan [38,45] Angola [5], and Indonesia [46]. This could probably be due to older children are in the transition life stage to adolescence when several unique challenges, including an increased body requirement for nutritional need are observed [47]. This also reveals that stunting is a long-lasting nutritional deprivation, developed a long period of time and hard to reverse once originated. The other possibility could be, nearly two-third of mothers were not attended formal education. These mothers might wrongly perceive, the older children get grown and wouldn’t be stunted.

Male primary school pupils were sevenfold at higher risk of stunting than females; which was supported by studies from the Ethiopia [14,48] and Tanzania [41]. In contrast, Studies from China [49], and Pakistan [50] revealed that females were more likely to be at risk than males. This might be males’ growth and development is more influenced by environmental and nutritional stress than females and thus, making males more likely to be affected by stunting [4]. This is also common and culturally known in Ethiopia that females are obliged to go home after school as they intend to learn in-home activities and to shoulder the responsibilities and share their mother’s home task. While the males are supposed to exercise, outdoor activities; like football playing and swimming at the river. And they might not give attention to the diet if they are not at home during the feeding time. It is also known that boys are known to have higher risk of stunting due to higher zinc requirement to meet their higher growth rate [51,52].

Age of the pupil and stunting has a positive correlation as age advances, the likelihood of being stunted increases. Similarly, male pupils are at high risk of being stunted due to different nutrient deficiencies [51,52]. Therefore, the Ethiopian ministry of health and education should give due attention to Zink supplementation, and school feeding should be a primary target of programs aiming at preventing stunting.

### Limitations of the study

The study was cross-sectional and thus cannot confer a causal relationship. We didn’t collect the morbidity and health services utilization data as the UNICEF conceptual framework considers these components for under-nutrition assessment. There might also be a potential bias Measurement bias might also occur, as not having a standardized height measurement. However, the data collectors trained on how to measure and pupils were removing all clothes and shoes except pants and shirts, which helped us to reduce the measurement bias.

## Conclusion

The prevalence of stunting was 15.13%, and the pupil’s age and sex were significantly associated with stunting. Therefore, this finding alerts the need to implement school health and nutrition programs to improve the nutritional status of school-age children in Bahir Dar city. The ministry of health and the respective zonal education department should strengthen and rapidly expanding the coverage of the school feeding program (Seqota declaration) to the city’s primary schools to improve school children’s health and nutrition status and to increase access to education. It is advisable to give Zink supplementation for male pupils and older age pupils. It is good to take due attention to extensively creating awareness of stunting and its consequences to the public primary school pupils by the health care workers, and through mass media to enhance the parents/caregivers’ understanding of undernutrition. Besides, large scale studies like the national nutritional surveys should contemplate school-age children as one element to evaluate their nutritional status regularly.

## Supporting information

S1 File Supporting information 2 (English version questionnaire). (PDF)

S2 File Supporting information 3 (SPSS data). (SAV)

## Abbreviations

AOR: Adjusted Odds Ratio
BMI: Body Max Index
CI: Confidence Interval
EDHS: Ethiopian Demographic and Health Survey
SD: Standard Deviation
WHO: World Health Organization

## References

[1] Hussein MD, Alonazi NA, Mohamed S. Prevalence of obesity, overweight, underweight, and stunting among school children in Argo city, Northern Sudan. Sudan J Paediatr. 2018;18(2):15–9. 10.24911/SJP.106-1544799078 30799893 PMC6378581

[2] Perignon M, Fiorentino M, Kuong K, Burja K, Parker M, Sisokhom S, et al. Stunting, poor iron status and parasite infection are significant risk factors for lower cognitive performance in Cambodian school-aged children. PLoS One. 2014;9(11):e112605. 10.1371/journal.pone.0112605 25405764 PMC4236074

[3] World health organization; Malnutrition. WHO. 2018;1–5.

[4] Tariku EZ, Abebe GA, Melketsedik ZA, Gutema BT. Prevalence and factors associated with stunting and thinness among school-age children in Arba Minch Health and Demographic Surveillance Site, Southern Ethiopia. PLoS One. 2018;13(11):e0206659. 10.1371/journal.pone.0206659 30388149 PMC6214544

[5] Oliveira D, Ferreira FS, Atouguia J, Fortes F, Guerra A, Centeno-Lima S. Infection by Intestinal Parasites, Stunting and Anemia in School-Aged Children from Southern Angola. PLoS One. 2015;10(9):e0137327. 10.1371/journal.pone.0137327 26371758 PMC4570803

[6] Black RE, Allen LH, Bhutta ZA, Caulfield LE, De Onis M, Ezzati M, et al. Maternal and child undernutrition: global and regional exposures and health consequences. The lancet. 2008;371(9608):243–60. 10.1016/S0140-6736(07)61690-0 18207566

[7] Bogale TY, Bala ET, Tadesse M, Asamoah BO. Prevalence and associated factors for stunting among 6–12 years old school age children from rural community of Humbo district, Southern Ethiopia. BMC public health. 2018;18(1):653. 10.1186/s12889-018-5561-z 29793479 PMC5968559

[8] Yeasmin S, Islam K. Prevalence and determinants of undernutrition among school age slum children in Dhaka City. Bangladesh J Nutr Health Sci. 2016;3(2):1.

[9] Yeasmin S, Islam K. A comparative study of health, nutritional status, and dietary pattern of primary school going and dropout slum children in Dhaka City, Bangladesh. Asian Journal of Medical Sciences. 2016;7(4):59–63.

[10] Mekonnen A, Tefera B, Woldehanna T, Jones N, Seager J, Alemu T, et al. Child nutritional status in poor Ethiopian households. 2005.

[11] Tadesse G. The prevalence of intestinal helminthic infections and associated risk factors among school children in Babile town, eastern Ethiopia. Ethiopian Journal of Health Development. 2005;19(2):140–7.

[12] Getaneh Z, Melku M, Geta M, Melak T, Hunegnaw MT. Prevalence and determinants of stunting and wasting among public primary school children in Gondar town, northwest, Ethiopia. BMC pediatrics. 2019;19(1):207. 10.1186/s12887-019-1572-x 31238889 PMC6591879

[13] Herrador Z, Sordo L, Gadisa E, Moreno J, Nieto J, Benito A, et al. Cross-sectional study of malnutrition and associated factors among school aged children in rural and urban settings of Fogera and Libo Kemkem districts, Ethiopia. PloS one. 2014;9(9). 10.1371/journal.pone.0105880 25265481 PMC4179248

[14] Tariku EZ, Abebe GA, Melketsedik ZA, Gutema BT. Prevalence and factors associated with stunting and thinness among school-age children in Arba Minch Health and Demographic Surveillance Site, Southern Ethiopia. PloS one. 2018;13(11). 10.1371/journal.pone.0206659 30388149 PMC6214544

[15] Nutrition Sco. Nutrition and the Post-2015 Sustainable Development Goals United nations system. october, 2014.

[16] Wikipedia F. List of cities and towns in Ethiopia. the free encyclopedia. 2016.

[17] Central statistical agency; Ethiopian demographic and health survey 2016: key indicators report. The DHS Program ICF. 2016;363:364.

[18] Bahir Dar city administration; Population and household survey. 2017/18.

[19] Bahir Dar city administration education department, annual report. 2018.

[20] Hailegebriel T. Undernutrition, intestinal parasitic infection and associated risk factors among selected primary school children in Bahir Dar, Ethiopia. BMC infectious diseases. 2018;18(1):394. 10.1186/s12879-018-3306-3 30103696 PMC6090691

[21] Webb P, Bhatia R. Manual: measuring and interpreting malnutrition and mortality. Nutr Serv WFP Rome. 2005;17.

[22] Bukania ZN, Mwangi M, Karanja RM, Mutisya R, Kombe Y, Kaduka LU, et al. Food insecurity and not dietary diversity is a predictor of nutrition status in children within semiarid agro-ecological zones in eastern Kenya. Journal of nutrition and metabolism. 2014;2014. 10.1155/2014/907153 25328691 PMC4195253

[23] Coates J, Swindale A, Bilinsky P. Household Food Insecurity Access Scale (HFIAS) for measurement of food access: indicator guide: version 3. 2007.

[24] Demographic E. Health Survey (EDHS) 2016: Key Indicators Report, Central Statistical Agency Addis Ababa, Ethiopia. The DHS Program ICF Rockville, Maryland, USA. 2016.

[25] Abate KH, Belachew T. Care and not wealth is a predictor of wasting and stunting of ‘The Coffee Kids’ of Jimma Zone, southwest Ethiopia. Nutrition and health. 2017;23(3):193–202. 10.1177/0260106017706253 28641475

[26] Getaneh Z, Melku M, Geta M, Melak T, Hunegnaw MT. Prevalence and determinants of stunting and wasting among public primary school children in Gondar town, northwest, Ethiopia. BMC Pediatr. 2019;19(1):207. 10.1186/s12887-019-1572-x 31238889 PMC6591879

[27] Bogale TY, Bala ET, Tadesse M, Asamoah BO. Prevalence and associated factors for stunting among 6–12 years old school age children from rural community of Humbo district, Southern Ethiopia. BMC Public Health. 2018;18(1):653. 10.1186/s12889-018-5561-z 29793479 PMC5968559

[28] Lisanu Mazengia A, Andargie Biks G. Predictors of Stunting among School-Age Children in Northwestern Ethiopia. J Nutr Metab. 2018;2018:7521751. 10.1155/2018/7521751 30327729 PMC6171210

[29] Tariku EZ, Abebe GA, Melketsedik ZA, Gutema BT. Prevalence and factors associated with stunting and thinness among school-age children in Arba Minch Health and Demographic Surveillance Site, Southern Ethiopia. PLoS One. 2018;13(11):e0206659. 10.1371/journal.pone.0206659 30388149 PMC6214544

[30] Grimes JET, Tadesse G, Gardiner IA, Yard E, Wuletaw Y, Templeton MR, et al. Sanitation, hookworm, anemia, stunting, and wasting in primary school children in southern Ethiopia: Baseline results from a study in 30 schools. PLoS Negl Trop Dis. 2017;11(10):e0005948. 10.1371/journal.pntd.0005948 28991894 PMC5633169

[31] Hussein MD, Alonazi NA, Mohamed S. Prevalence of obesity, overweight, underweight, and stunting among school children in Argo city, Northern Sudan. Sudanese journal of paediatrics. 2018;18(2):15. 10.24911/SJP.106-1544799078 30799893 PMC6378581

[32] Mohamed S, Hussein MD. Prevalence of thinness, stunting and anemia among rural school-aged sudanese children: a cross-sectional study. Journal of Tropical pediatrics. 2015;61(4):260–5. 10.1093/tropej/fmv028 25896992

[33] Adedeji IA, Bashir MF, Shwe DD, John C. Prevalence and correlates of stunting among the school-age population in North-Central Nigeria. Pan Afr Med J. 2018;31:170. 10.11604/pamj.2018.31.170.15763 31086623 PMC6488238

[34] Esfarjani F, Roustaee R, Mohammadi F, Esmaillzadeh A. Determinants of stunting in school-aged children of tehran, iran. Int J Prev Med. 2013;4(2):173–9. 23543188 PMC3604849

[35] Dong Y, Bennett K, Jan C, Dong B, Zou Z, Hu P, et al. Subnational variation of stunting, wasting and malnutrition in Chinese primary-school children between 2010 and 2014: urban–rural disparity. Public health nutrition. 2019;22(11):2043–54. 10.1017/S1368980019000235 30827292 PMC10273615

[36] Qin Y, Melse-Boonstra A, Zhao J, Wu M, Hu X, Kok FJ. Stunting and zinc deficiency among primary school children in rural areas with low soil zinc concentrations in Jiangsu Province, China. Asia Pacific journal of clinical nutrition. 2009;18(1):15. 19329390

[37] Wu H, Li H, Zong X. The prevalence of overweight, obesity and stunting in school children aged 6–19 years in Beijing, China. Annals of human biology. 2016;43(6):505–9. 10.3109/03014460.2015.1107129 26464071

[38] Mushtaq MU, Gull S, Khurshid U, Shahid U, Shad MA, Siddiqui AM. Prevalence and socio-demographic correlates of stunting and thinness among Pakistani primary school children. BMC Public Health. 2011;11(1):790. 10.1186/1471-2458-11-790 21988799 PMC3209698

[39] McDonald CM, Baylin A, Arsenault JE, Mora-Plazas M, Villamor E. Overweight is more prevalent than stunting and is associated with socioeconomic status, maternal obesity, and a snacking dietary pattern in school children from Bogota, Colombia. J Nutr. 2009;139(2):370–6. 10.3945/jn.108.098111 19106320 PMC2646207

[40] Lisanu Mazengia A, Andargie Biks G. Predictors of Stunting among School-Age Children in Northwestern Ethiopia. Journal of nutrition and metabolism. 2018;2018. 10.1155/2018/7521751 30327729 PMC6171210

[41] Altare C, Delbiso TD, Mutwiri GM, Kopplow R, Guha-Sapir D. Factors Associated with Stunting among Pre-school Children in Southern Highlands of Tanzania. J Trop Pediatr. 2016;62(5):390–408. 10.1093/tropej/fmw024 27122480 PMC5040832

[42] Adenuga W, Obembe T, Odebunmi K, Asuzu M. prevalence and determinants of stunting among primary school children in rural and urban communities in Obafemi Owode local government area, Southwestern Nigeria. Annals of Ibadan postgraduate medicine. 2017;15(1):7–15. 28970765 PMC5598447

[43] Senbanjo IO, Oshikoya KA, Odusanya OO, Njokanma OF. Prevalence of and risk factors for stunting among school children and adolescents in Abeokuta, Southwest Nigeria. Journal of health, population, and nutrition. 2011;29(4):364. 10.3329/jhpn.v29i4.8452 21957675 PMC3190367

[44] Al-Saffa AJ. Stunting among primary-school children: a sample from Baghdad, Iraq. East Mediterr Health J. 2009;15(2):322–9. 19554978

[45] Khuwaja S, Selwyn BJ, Shah SM. Prevalence and correlates of stunting among primary school children in rural areas of southern Pakistan. Journal of tropical pediatrics. 2005;51(2):72–7. 10.1093/tropej/fmh067 15677373

[46] Yasmin G, Kustiyah L, Dwiriani CM. Risk factors of stunting among school-aged children from eight provinces in Indonesia. Pakistan Journal of Nutrition. 2014;13(10):557.

[47] Akseer N, Al‐Gashm S, Mehta S, Mokdad A, Bhutta ZA. Global and regional trends in the nutritional status of young people: a critical and neglected age group. Annals of the New York Academy of Sciences. 2017;1393(1):3–20. 10.1111/nyas.13336 28436100

[48] Ethiopian statistical agency. Ethiopian demographic and health survey 2016.

[49] Wu H, Li H, Zong X. The prevalence of overweight, obesity and stunting in school children aged 6–19 years in Beijing, China. Ann Hum Biol. 2016;43(6):505–9. 10.3109/03014460.2015.1107129 26464071

[50] Khuwaja S, Selwyn BJ, Shah SM. Prevalence and correlates of stunting among primary school children in rural areas of southern Pakistan. J Trop Pediatr. 2005;51(2):72–7. 10.1093/tropej/fmh067 15677373

[51] Hotz C, Brown KH. International Zinc Nutrition Consultative Group (IZiNCG) technical document 1: assessment of the risk of zinc deficiency in populations and options for its control. 2004. 18046856

[52] Qin Y, Melse-Boonstra A, Zhao J, Wu M, Hu X, Kok FJ. Stunting and zinc deficiency among primary school children in rural areas with low soil zinc concentrations in Jiangsu Province, China. Asia Pacific journal of clinical nutrition. 2009. 19329390

