# Supplementary material for: Prevalence of stunting and associated factors among public primary school pupils of Bahir Dar city, Ethiopia: School-based cross-sectional study
Source: PLoS One. 2021 Apr 12;16(4):e0248108. doi: 10.1371/journal.pone.0248108 (PMC8041191; doi:10.1371/journal.pone.0248108)
Supplement: S1 File — (PDF) [file pone.0248108.s001.pdf]

English Questionnaire

PART-1: Sociodemographic characteristics of the respondents

S. No	**Variables**	**Categories**	**Skip**
101	Age of the pupil	1. ---------------years	
102	Sex of the pupil	1. Male<br>2. Female	
103	Weight of the pupil	____________kg	
104	Height of the pupil	____________meter	
105	Mid upper arm circumference	____________centimeter	
106	Body Mass Index	____________kg/m^2	
107	Religion of the parents	1. Orthodox<br>2. Muslim<br>3. Catholic<br>4. Protestant<br>5. Others (if any specify) __________	
108	Family size	_________	
109	Father's occupation	1. Merchant<br>2. Government employee<br>3. Self-employee<br>4. Others (if any, specify) _________	
110	Mother's occupation	1. Merchant<br>2. Government employee<br>3. Self-employee<br>4. Others (if any, specify) _________	
111	Education status of the father	1. Unable to read and write<br>2. Able to read and write only<br>3. Primary education attended<br>4. Secondary education attended<br>5. Higher education attended	

	Education status of the mother	1. Unable to read and write<br>2. Able to read and write only<br>3. Primary education attended<br>4. Secondary education attended<br>5. Higher education attended	
112	Monthly income of the family (in birr)	____________	
113	Number of children in the household	____________	
114	The birth order of the child	____________	
115	Do you have backyard or guarding	1. Yes<br>2. No	
116	Does the mother participate in child feeding	1. Yes<br>2. No	
117	How food is obtained in the family	1. Own production<br>2. Purchases<br>3. Food aid	
118	Do you warry that the household doesn’t have enough food	1. Yes<br>2. No	
119	If the answer for question 118 is ‘yes’, how many times	1. Always<br>2. Usually<br>3. Sometimes	
120	If the family got shortage of food, what is your measurement?	1. Share with neighbors/relatives<br>2. Debt<br>3. Work hard<br>4. Do nothing<br>5. Others (if any, specify) ________	
121	In your judgement is there anyone who didn’t get enough food in your family?	1. Yes<br>2. No	
	If your answer to question 121 is ‘yes’ how many times?	1. Always<br>2. Usually	

		3. Sometimes	
122	Did you face shortage of food?	1. Yes<br>2. No	
123	If your answer to question 122 is 'yes', what is the reason?	1. Diverse<br>2. Fool about<br>3. Income shortage<br>4. Lack of peace in the country<br>5. Others (if any, specify) ________	
124	Do you hear about variety of food?	1. Yes<br>2. No	
125	If your answer to question 124 is 'yes', who tell you?	1. Health care provider<br>2. Family member<br>3. Television<br>4. Radio<br>5. All<br>6. Others (if any, specify) ________	
126	Do you think variety of food good for child?	1. Yes<br>2. No	
127	If your answer to question 126 is 'yes', how many times?	1. Sometimes<br>2. As occasional<br>3. $\geq$ 3 days per week	
128	What is your first choose if your baby become ill?	1. Health facility<br>2. Traditional medicine<br>3. Holy water<br>4. Others (if any, specify) ________	
129	Any child that has any of this chronic disease? (DM, Hypertension, Asthma)	1. Yes<br>2. No	
	What is your child condition?	1. Healthy<br>2. Ill	
